# Safety of corticosteroid therapy in sarcoidosis treatment

**DOI:** 10.3389/fdsfr.2023.1319931

**Published:** 2023-12-14

**Authors:** Alessandro Di Marco Berardino, Federico Mei, Lina Zuccatosta

**Affiliations:** ^1^ Respiratory Diseases Unit, Department of Internal Medicine, Azienda Ospedaliero Universitaria delle Marche, Ancona, Italy; ^2^ Department of Biomedical Sciences and Public Health, Polytechnic University of Marche, Ancona, Italy

**Keywords:** sarcoidosis, treatment, corticosteroids, diabetes, obesity, cardiovascular disease, osteoporosis, systemic effects

## Abstract

**Introduction:** Sarcoidosis is a multisystemic granulomatous disease of unknown origin and unpredictable cause, characterized by a dysregulated immune response. If histopathological hallmark is represented by the presence of non-caseating granulomas, clinical manifestations are variable and symptoms are not specific, and they depend on organs affected. Although thoracic involvement (lung and mediastinum) is the most common clinical manifestation, any organ can be virtually affected.

**Methods:** This paper is structured as a narrative review. A literature search was performed in four electronic databases (Pubmed, Cochrane, Scopus, and Ovid Medline) and Google from inception until February 2023 for relevant studies, meta-analyses, and reviews on corticosteroids’ adverse events in sarcoidosis. English language only papers were included.

**Discussion:** Although antimetabolites (such as Methotrexate) and immunosuppressant agents can be used as alternative therapy in refractory cases, traditionally systemic glucocorticoids represent the first choice for sarcoidosis treatment. However, their use is still debated, due to potential adverse effects, leading to a wide spectrum of complications particularly in patients who required long-term therapy. Hence, this article aims to provide a comprehensive updated review on the safety profile of glucocorticoid treatment in patients with sarcoidosis and their systemic effects.

**Conclusion:** corticosteroids remain the first choice in Sarcoidosis, however, due to numerous side effects, dose and duration of treatment should be carefully adjusted and monitored by clinicians.

## Introduction

Sarcoidosis was first described in 1869 by Dr. Jonathan Hutchinson, an English dermatologist, with a case of cutaneous sarcoidosis in a coal worker (Drent et al., 2021a).

It is a multisystemic inflammatory disease of unknown cause that is characterized by a dysregulated immune response to certain environmental antigens, resulting in sustained granulomatous inflammation and failure to clear the offending antigens. Other conditions, such as infectious disorders and cancer, have also been shown to be associated with a granulomatous reaction, mimicking sarcoidosis. In addition, certain drugs may also induce sarcoid-like reactions indistinguishable from sarcoidosis (Bonifazi et al., 2015; Bonifazi et al., 2021; Tana et al., 2022).

The clinical picture is usually wide and variable, depending on the organ(s) primarily affected, with non-specific symptoms of respiratory involvement (e.g., dry cough, dyspnoea, fatigue and chest pain) representing the most common clinical presentation.

The prevalence ranges from 1 to 5 cases per 100,000 in Japan, Taiwan and South Korea to 140–160 cases per 100,000 in Sweden and Canada with the highest mortality rate observed in Black Americans (Arkema and Cozier, 2020).

Usually, patients with an acute onset as in Lofgren’s syndrome (e.g., bilateral hilar lymph adenopathy, erythema nodosum, fever and arthritis) have a good prognosis, whereas the presence of organ involvement (cardiac, neurologic, renal and pulmonary involvement with fibrosis) is associated with increased morbidity and mortality as well as chronic course, and indicates the need for treatment (Tana et al., 2022).

There are, generally, two indications for the treatment of sarcoidosis, being the development of a dangerous health situation (heart, lung, brain involvement) and a significant worsening of quality of life.

When therapy is indicated, corticosteroids (CS) are usually the first-choice drugs, because they work more reliably and more rapidly than all other alterative agents (Judson, 2016; Baughman et al., 2017).

However, although a recent consensus statement from sarcoidosis experts still endorses CS as the primary treatment for sarcoidosis, considerable controversies and concerns remain when CS are used in sarcoidosis treatment related to a high risk of toxicity (Baughman et al., 2021). Numerous and significant adverse drug reactions (ADRs) can be associated with CS use, particularly in patients with long-term therapy, being able to potentially involve different organs including bone and muscles, endocrine and cardiovascular system, skin, eyes and mood changes (Table 1). Afterward, CS are often discontinued and alternative corticosteroid-sparing or corticosteroid-replacing (Methotrexate, Azathioprine, TNF-a inhibitors) are required (Oray et al., 2016; Drent et al., 2021b).

**TABLE 1:** Long-term side effects of glucocorticoids.

System affected	Manifestations
Endocrine System	Diabetes mellitus type II, hyperglycaemia, dyslipidaemia, obesity and weigh gain, adrenal and growth suppression, Cushingoid features, secondary hyperparathyroidism
Skin	Dermatoprosis, ecchymoses, purpura, skin atrophy, striae, telangiectasia, hematoma, acne, hirsutism, hair loss
Musculoskeletal System	Osteoporosis, fracture, avascular necrosis, myopathy
Cardiovascular system	Hypertension, congestive heart failure, coronary artery disease, cerebrovascular accident, atrial fibrillation, acute myocardial infarction
Gastrointestinal system	Gastritis, peptic ulcers with or without bleeding, pancreatitis, hepatic steatosis, perforations
Behaviour	Fatigue, depression, euphoria, irritability, anxiety, cognitive impairment, delirium, dementia
Sleep disorders	Sleep apnoea syndrome (unclear)
Haemopoietic system	Suppression of cell-mediated immunity, polycythemia

The clinical manifestations of these iatrogenic conditions (fatigue, weakness and muscles pain) may be difficult to distinguish from sarcoidosis-associated symptoms. Timing, pattern of illness, results of investigations and drug rechallenging might be helpful to detect suspected ADRs (Edwards and Aronson, 2000). Nowadays pharmacogenomic (PGx) testing use is rising and can be applied to identify patients with genetic variants, predicting, therefore, treatment outcomes and potential harms (Hahn and Roll, 2021).

It is also worth mentioning that higher dose of CS has been linked to increased number of non-sarcoidosis-related emergency department visits compared to patients with lower cumulative CS exposure. Harper et al. showed that new steroid-related comorbidities and poor outcomes can be predicted by low income in US patients with sarcoidosis, following by current or past medication use, higher age and longer duration of symptoms (Oray et al., 2016; Ha et al., 2020).

Hence, this review aims to describe main ADRs of sarcoidosis treatment and comorbidities, particularly focusing on corticosteroids toxicity according to organ involvement.

## Methods

A literature search was performed in four electronic databases (Pubmed, Cochrane, Scopus, and Ovid Medline) and Google from inception until February 2023 using following terms: “sarcoidosis,” “corticosteroids treatment,” “glucocorticoids,” “steroids,” “systemic effects,” “adverse events,” “treatment side effects,” “complications,” “endocrine system,” “diabetes,” “obesity,” “adrenal gland,” “dyslipidemia,” “musculoskeletal system,” “bone metabolism,” “skin,” “cardiovascular disease,” “gastrointestinal system,” “neuropsychiatric effects,” “behavior,” “sleep disorders,” “hematopoietic system.” Exclusion criteria included commentaries and non-English language articles. Titles and abstracts were first examined to determine their relevance to the review. Duplicate articles between databases were initially identified and appropriately excluded. For this narrative review, an ethics committee approval was not required.

## Endocrine system and metabolic conditions

### Type 2 diabetes

Several studies have shown that type-2 diabetes (T2D) is more prevalent in patients with sarcoidosis compared with age- and sex-matched controls (Brito-Zeron et al., 2018; Benmelouka et al., 2021). The mechanism by which glucocorticoids cause hyperglycemia is multifactorial, including augmentation of hepatic gluconeogenesis, inhibition of glucose uptake in adipose tissue, and alteration of receptor and post-receptor functions (Schacke et al., 2002).

However, to clarify the extent of contribution of CS in increasing risk of T2D in sarcoidosis patients, Entrop et al. performed a large cohort study including untreated and CS-treated subjects with sarcoidosis and general population matched by age, sex and region of residence. The T2D rate was higher in both the CS-treated (HR: 2.44) and untreated (HR: 1.44) sarcoidosis groups compared with the general population. The risk was highest in males who received CS at sarcoidosis diagnosis and in older patients (Entrop et al., 2021).

Furthermore, the presence and severity of metabolic disorders have also been suggested to impact on the severity of pulmonary sarcoidosis. In a retrospective cohort study, 220 sarcoidosis patients were classified into diabetic and non-diabetic groups and several metabolic conditions (lipid status, body mass index - BMI, glycemic control) were assessed. Results from this study showed diabetic patients having lower total lung capacity (TLC) and diffusion capacity of the lung for carbon monoxide (DLCO) in comparison with non-diabetic subjects. In addition, female, non-smoker and morbidly obese (BMI >35) diabetic patients had significantly lower DLCO than their non-diabetic matching people, suggesting metabolic disorders may be an independent risk factor for worse pulmonary sarcoidosis.

### Obesity

The development of Cushingoid features (redistribution of body fat with truncal obesity, buffalo hump, and moon face) and weight gain are dose- and duration-dependent and can develop within the first 2 months of therapy, being also iatrogenic Cushing’s syndrome a marker for patients at a higher risk of cardiovascular disease (Boumpas et al., 1993; Fardet et al., 2012).

Factors that may also contribute to raised weight include an increased appetite, a common side effect of glucocorticoid therapy, and an increase in food intake for symptomatic relief in patients with gastropathy or peptic ulcer disease because of CS therapy. Recent findings showed that obesity can be both a consequence of sarcoidosis treatment, and a contributor to disease risk likely through the pro-inflammatory environment of obesity. In fact, in obese patients adipose tissue is rich in innate and adaptive immune cells and it tends to switch towards a T-helper 1 proinflammatory phenotype (Kanneganti and Dixit, 2012; Schipper et al., 2012; Ellulu et al., 2017).

### Dyslipidemia

The effects of CS on lipidic profile are controversial, being literature data showing possible beneficial effects of glucocorticoids on dyslipidemia.

Findings from observational studies suggested that adverse effects of CS on the lipid profile are dose-dependent, occurring only at prednisone doses greater than 10 mg/day (Leong et al., 1994).

Moreover, corticosteroids may act by leading sequentially to peripheral insulin resistance, hyperinsulinemia, and increased hepatic very low-density lipoprotein (VLDL) synthesis.

However, glucocorticoid-induced reduction in corticotropin (ACTH) release also contributes to the lipid changes, reducing total and low-density lipoprotein (LDL) cholesterol and triglycerides (Berg and Nilsson-Ehle, 1996).

### Adrenal suppression

Administration of exogenous CS can suppress the hypothalamic-pituitary-adrenal axis (HPA) and it has been shown that the long term of steroids use is associated with adrenal gland suppression with significant individual variation in response. Patients who are using CS chronically may suffer hypotension and cardiovascular collapse if steroids are suddenly withdrawn or tapered too quickly as a result of the adrenal gland suppression.

Although normalization of the adrenal gland function after CS withdrawal may require up to 9 months, most patients have a normal function within the first month depending on the duration and dosage of CS and speed of taper (Ahmet et al., 2019).

## Skin

Corticosteroids act on keratinocytes and prevent the secretion of collagen and hyaluronic acid by fibroblasts in the dermis, tampering with cell proliferation and ultimately leading to thinning of the skin (Da Silva et al., 2006).

Dermatoporosis, comparable to osteoporosis in the elderly, is characterized by chronic skin insufficiency and fragility. Main findings of dermatoporosis are thinning, telangiectasia, and hematoma of the skin that becomes lacerated with poor healing in advanced stages.

The ecchymoses or purpura associated with CS use often affect the sun-exposed areas of the dorsum of the hand and forearm and are not accompanied by palpable swelling.

The prevalence of these dermatological complications is increased with long-term and higher dose treatment, also raising the risk of catabolic effects of CS, which may be associated with atrophy, striae, delayed wound healing, steroid acne, hirsutism, and hair loss (Kaya and Saurat, 2007).

## Muscoloskeletal system

Direct effects of sarcoidosis on the calcium metabolism are well-described, particularly hypercalcaemia, which is usually due to the increased activity of macrophages, with altered high levels of calcitriol and low levels of 25-hydroxyvitamin D (Gianella et al., 2020).

Clinical manifestations of hypercalcaemia are systemic symptoms such as nausea, vomiting, confusion, delirium, coma, and spasms and organ manifestations such as heart arrhythmias, nephrolithiasis, and renal failure (Ponce and Gujral, 2004; Tebben et al., 2016).

Less clear is the relationship between sarcoidosis (prior to steroid therapy) and osteoporosis; while some studies show a decrease in bone mineral density (Ponce and Gujral, 2004; Ungprasert et al., 2017a), others have failed to find a correlation between sarcoidosis and osteoporosis among untreated patients (Heijckmann et al., 2007; Bolland et al., 2015; Bours et al., 2016). One study describes an augmented risk for fractures (23.5%) without a change in bone mineral density, suggesting that other independent factors are involved (Saidenberg-Kermanac’h et al., 2014).

These data are more evident in postmenopausal women when compared with premenopausal women, suggesting a non-neglectable role of sex hormones (Rizzato et al., 1992; Adler et al., 2003a).

Moreover, in a systematic review and meta-analysis, Yong and others failed to find an increased risk of fracture and bone mineral loss. However, considering the high heterogeneity of the study, they still suggested screening for osteoporosis (Yong et al., 2019).

Regarding bone health, pharmacological treatment of sarcoidosis, especially corticosteroid therapy, plays a pivotal role.

According to guidelines, these drugs represent the first-line therapeutic option for patients and remain the most common strategy for treatment (Crouser et al., 2020; Baughman et al., 2021; Thillai et al., 2021).

Moreover, the effects on bones of long-term use of steroids in chronic illness have been studied since 1940 and are now well-known, with a large portion of these subjects developing osteoporosis. In these subjects, osteoporosis usually begins quickly (approximately 3 months after treatment initiation) but reduces its progression over time (Sweiss et al., 2011; Cosman et al., 2014; Messina et al., 2022).

The risk of fracture is influenced by both the dose and duration of steroid therapy (Oray et al., 2016); it declines after cessation of oral corticosteroid treatment, and a low dose for a prolonged time results in an increased risk of fracture (Van Staa et al., 2000; Abtahi et al., 2022).

Among patients with chronic respiratory diseases generally treated with steroids (such as chronic obstructive pulmonary disease [COPD]), osteoporosis is more common than in the general population (Incalzi et al., 2000; Adler et al., 2003b).

Moreover, compared with patients affected by sarcoidosis, COPD patients have higher bone mineral density (BMD) (Adler et al., 2003a), suggesting an incremental role in bone turnover in this disease.

Fracture risk is increased in all patients with chronic diseases treated with long-term steroids, such as lupus, rheumatoid arthritis, inflammatory bowel disease, and multiple sclerosis (Balasubramanian et al., 2018).

For sarcoidosis, Heijckmann et al. have found vertebral deformities suggestive of fractures in 20% of patients despite a normal BMD, according to increased bone turnover evaluated using carboxy-terminal cross-linked telopeptide of type I collagen (ICTP) and the bone formation marker, serum procollagen type I amino-terminal propeptide (PINP). According to these data, BMD failed in evaluating bone turnover. ICTP levels have a significant relationship with the soluble interleukin-2 receptor (sIL2R) and angiotensin-converting enzyme (ACE), used as parameters of disease activity (Heijckmann et al., 2007).

This group of patients was followed for 4 years; the BMD remained the same, but the study showed a significant increase in the prevalence of vertebral deformities (20%–32%), and the risk of new or progressive vertebral deformity fracture was significantly influenced by the T-score of the femoral neck at baseline and familiarity (mother) with a history of hip fracture. This evidence seems to confirm the poor capacity of BMD to predict fractures (Heijckmann et al., 2008).

A large retrospective cohort study by Bours et al. studied 5,722 patients affected by sarcoidosis *versus* 28,704 controls matched for age and sex. Patients with sarcoidosis were more likely to have used medical drugs in the previous 6 months (including glucocorticoids, benzodiazepines, antidepressants, antidiabetics and anti-osteoporosis therapy). Among them, 406 have had at least one fracture. In particular, the risk of vertebral fractures was significantly increased in this group of patients; on the contrary, the risk for non-vertebral fractures was decreased. Regarding therapy, the use of steroids in the previous 6 months had a significant relationship with an increased risk of fracture and osteoporosis. Moreover, the risk remained higher with low doses of corticosteroid therapy (less than 5 mg of prednisone) and with the lowest cumulative doses (less than 1,820 mg) (Bours et al., 2016).

A more recent case-control study based on the Danish National Hospital Discharge Registry (NHDR) showed an increased risk of osteoporotic fractures in a group of 124 patients affected by sarcoidosis in therapy with glucocorticoids, but only in patients with a cumulative dose between 1 mg and >10 mg of prednisolone equivalent; surprisingly, when the dose was less than 1 mg or between 5 and 9.9 mg, there was no difference with the control group (Oshagbemi et al., 2017).

A recent work performed in Italy by Caffarelli et al. evaluated 252 patients and 250 healthy controls, matched for sex and age. Significant differences between the two groups were found regarding BMD, but only for the lumbar spine and total hip BMD. The BMD values in all skeletal sites showed a significant association with the lung diffusion capacity of carbon dioxide (DLco). Fragility fractures are more frequent in the sarcoidosis (30.6%) group than in controls (12.3%), and the most common site is represented by vertebrae (in particular T6–T7 and T11). Furthermore, subjects with three or more vertebral fractures have a lower spirometric function, particularly FEV1, FVC and DLco (Caffarelli et al., 2022).

Regarding the musculoskeletal apparatus, muscle and articulations could be a target of sarcoidosis. In fact, acute and chronic arthritis and myopathy (nodular type, chronic myopathic type, acute myositic type and smouldering type) are relatively common presentations of the disease (Hasbani et al., 2022).

Excluding osteoporosis, there are few studies regarding other musculoskeletal adverse reactions to steroids in sarcoidosis (Drent et al., 2022; Tana et al., 2022). A more common complication is represented by myopathy and avascular necrosis of bone (in particular, osteonecrosis of the femoral head). This is similar to other disease treated with long-term steroid therapy such as inflammatory bowel disease (Giraud et al., 2021; Melani et al., 2021).

## Cardiovascular system

The impact on the cardiovascular system (CVS) of immunomodulated diseases treated with prolonged steroid therapy is well-known (Pujades-Rodriguez et al., 2020; Galiuto and Volpe, 2021; Giraud et al., 2021; Melani et al., 2021; Drent et al., 2022; Tana et al., 2022).

In a cohort study by Pujades-Rodriguez et al., a population of 87,794 patients affected by giant cell arteritis, polymyalgia rheumatica, inflammatory bowel disease, rheumatoid arthritis, systemic lupus erythematosus or vasculitis without any history of cardiovascular disease (CVD) had an increased risk of all-cause CVD not only for high doses of steroids (more than 25 mg prednisolone-equivalent) but also for lower doses (less than 5 mg prednisolone-equivalent) (Pujades-Rodriguez et al., 2020).

The influence of immune-mediated diseases on the CVS was confirmed by Zoller et al. in a large study performed in Sweden, where sarcoidosis was included (Zoller et al., 2012).

Yafasova et al., in a cohort of 364 subjects affected by sarcoidosis (112 treated with steroids), confirmed a higher risk for heart failure (HF) and other cardiac outcomes such as ventricular arrhythmia, a pacemaker, or an implantable cardioverter defibrillator (ICD) implantation (Yafasova et al., 2020).

Regarding therapy, Ungprasert et al., based on 345 patients, confirmed an increased risk for CVD, particularly congestive heart failure (CHF), coronary artery disease, cerebrovascular accident and atrial fibrillation. However, the hazard ratio between treated and untreated patients (considering not only steroids but any immunosuppressive therapy) was not statistically different (Ungprasert et al., 2017b; Rossides et al., 2022).

In recent work, Rossides et al. reported a 2.43 Hazard Ratio for HF when sarcoidosis was present; it was higher in the first 2 years of follow-up and in people without a history of ischaemic heart disease. In contrast to the work of Ungprasert et al., they demonstrated the influence of steroid therapy. A cumulative defined daily dose of systemic steroids (DDDs; one DDD prednisolone = 10 mg) higher than 300 DDDs was associated with an increased risk of HF compared to 150 or lower DDDs (Rossides et al., 2022).

Regarding the risk of acute myocardial infarction, the same group, in another publication, found a higher risk with respect to the general population. However, the highest HR was observed in patients treated with an immunosuppressant drug at diagnosis. Post-hoc analyses showed a 50% higher risk of acute myocardial infarction in those treated during follow-up, but there was no relationship with dose (Rossides et al., 2021).

The mechanisms of these manifestations are still debated. However, a secondary role is played by the side effects of prolonged therapy with steroids, such as diabetes, arterial hypertension or weight gain, which represent *per se* important risk factors for CVD (Galiuto and Volpe, 2021; Rossides et al., 2021).

## Gastrointestinal system

The involvement of the gastrointestinal system (GIS) in sarcoidosis has been reported, and the effects of chronic steroids on the GIS are well-known (Ghrenassia et al., 2016; Sollors et al., 2022; Cho et al., 2023). Gastritis, peptic ulcers with or without bleeding, perforations, pancreatitis, and hepatic steatosis, are the most described side effects; furthermore, they increase the burden of the disease (Drent et al., 2022; Tana et al., 2022).

A transnational study performed by Drent et al. in the Netherlands, United Kingdom, and the United States of America studied gastrointestinal side effects of pharmacotherapy in sarcoidosis. A survey described the self-reported symptoms of 937 patients; 282 were untreated, and 416 were treated with prednisone alone or in combination with other drugs (methotrexate, TNF-alpha inhibitors, azathioprine, hydoxychloroquine and mycophenolate mofetil). The most common symptoms with prednisone alone were increased appetite (OR 9.26) and weight gain (OR 5.68). Still, surprisingly, when prednisone was combined with methotrexate, these side effects remained significantly higher when compared with methotrexate alone. Interestingly, with combination therapy, nausea increased with respect to prednisone monotherapy (Drent et al., 2020). In this ambit, nutrition could play a role (Bast et al., 2018).

## Neuropsychiatric effects

Long-term therapy with steroids can also have neurological and neuropsychiatric effects; fatigue, depression, euphoria, irritability, and anxiety and manifestations such as psychosis, delirium and dementia have been described (Tana et al., 2022).

Cox et al. evaluated the quality of life of 120 patients. Depression was assessed using the Centre for Epidemiologic Studies Depression Questionnaire. Stress was assessed by the Perceived Stress Scale. The prevalence was 66% for depression and 55% for stress. However, they failed to find differences between groups treated or untreated with steroids, despite the first group having a significantly worse quality of life (Cox et al., 2004).

Judson et al. compared two groups of patients treated with low or high doses of prednisone. A sarcoidosis assessment tool for fatigue, daily activities and satisfaction were significantly increased in the first group (Judson et al., 2015).

Among general symptoms, weakness and fatigue are extensively studied in sarcoidosis, and most authors agree with a multifactorial origin of these symptoms, in which steroids could have a direct and indirect effect (de Kleijn et al., 2009; Drent et al., 2012; Atkins and Wilson, 2017; Hendriks et al., 2018).

Fleisher et al., in their interesting study on factors associated with fatigue in sarcoidosis, showed an association between fatigue and medications; in particular, subjects who had received at least two medications (prednisolone and methotrexate) had increased fatigue levels, with respect to untreated patients or those with only one kind of medication (Fleischer et al., 2014).

However, steroids influenced fatigue indirectly through important factors such as weight gain and muscular weakness, which were, in turn, influenced by steroid therapy (Drent et al., 2012). For these reasons, a definitive cause–effect explanation is, at present, very difficult.

## Sleep disorders

Body mass index and obstructive sleep apnoea syndrome are strictly connected with fatigue and weight gain (Drent et al., 2000). The incidence of OSA and other sleep disorders is higher among patients with sarcoidosis; however, the role of therapy remains unclear (P et al., 2013; Verbraecken et al., 2004). For example, Turner et al. evaluated 83 patients with sarcoidosis; an increased risk of OSA was found with respect to the general population. However, among patients who underwent steroid treatment, despite increased body mass index, there was not a higher risk of OSA (Turner et al., 1997).

Bingol et al. compared two groups of patients, 15 with parenchymal involvement (7 treated with steroids) and 14 without (only 1 in therapy) and found significantly higher values of the apnoea–hypopnea index (AHI) and oxygen desaturation index (ODI) in the first group, suggesting a possible role of therapy. However, data are inconclusive, and the respective influence of lung involvement and medications on respiratory nocturnal parameters remains under debate (Bingol et al., 2015).

## Haemopoietic system

Corticosteroids increase hemoglobin and red cell content of blood, possibly by retarding erythrophagocytosis. This effect is demonstrated by the occurrence of polycythemia in Cushing disease and mild normochromic anemia in Addison disease. Corticosteroids also affect circulating white blood cells. Glucocorticoid treatment results in increased polymorphonuclear leukocytes in blood as a result of increased rate of entrance from the marrow and a decreased rate of removal from the vascular compartment. In contrast, the lymphocytes, eosinophils, monocytes, and basophils decrease in number after administration of glucocorticoids. A single dose of cortisol results in a 70% decrease in lymphocytes and a 90% decrease in monocytes, occurring 4–6 h after treatment and persisting for about 24 h. Cell numbers then rise 24–72 h after treatment (Pountain et al., 1993). The decrease in lymphocytes, monocytes and eosinophils is generally thought to be a consequence of redistribution of these cells, although certain lymphocytes also undergo glucocorticoid-induced apoptosis (Schwartzman and Cidlowski, 1994). T lymphocytes are more sensitive to glucocorticoid-induced apoptosis than are B lymphocytes, and T-cell subpopulations differ in their glucocorticoid sensitivity. A decrease in basophils occurs by an unknown mechanism.

## Conclusion

Sarcoidosis is an inflammatory disorder of unknown origin with multisystemic involvement.

When treatment is required, corticosteroids remain the first step of choice, due to their rapid action. However, corticosteroids have numerous side effects, which can reduce the quality of life in sarcoidosis patients, especially in case of prolonged use. This aspect could be evaluated by using established questionnaires specific for sarcoidosis.

Questionnaire on general health status (like Short Form-36) or dedicated only to respiratory symptoms (like St George’s Respiratory Questionnaire) could be less specific in this case (Baughman et al., 2021).

Others, like Sarcoidosis Activity and Morphology Instrument (CSAMI), Sarcoidosis Activity and Severity Index (SASI) or the Sarcoidosis Severity Tool (which is used to evaluate the severity of pulmonary disease) are organ-specific and they are not able to detect the impact of the disease from the patient’s perspective (Wasfi et al., 2006; Baughman et al., 2008; Rosenbach et al., 2013).

King’s Sarcoidosis Questionnaire (KSQ) could represent a useful solution for this aspect.

KSQ is a health status questionnaire developed specifically for patients with sarcoidosis. It is divided in five different modules (General health status, Lung, Skin, Eye, Medications). It is simple to administer for clinicians and easy to complete for patients. The validity of the Lung module was higher when compared with St George’s Respiratory Questionnaire (Patel et al., 2013).

In conclusion Guidelines recommend that the treatment should be planned with patients, starting with low-to medium-dose glucocorticoid (5–10 mg a day), adjusting dose and duration of maintenance based on the efficacy/side-effects balance, but for patients without significant impairment of quality of life (valuated with validated methods), no glucocorticoid treatment could be recommended (Baughman et al., 2021).
